# Whole blood microRNAs as potential biomarkers in post-operative early breast cancer patients

**DOI:** 10.1186/s12885-018-4020-7

**Published:** 2018-02-06

**Authors:** Marianna Alunni-Fabbroni, Leonie Majunke, Elisabeth K. Trapp, Marie Tzschaschel, Sven Mahner, Peter A. Fasching, Tanja Fehm, Andreas Schneeweiss, Thomas Beck, Ralf Lorenz, Thomas W. P. Friedl, Wolfgang Janni, Brigitte Rack, on behalf of the SUCCESS Study Group

**Affiliations:** 1 Department of Gynecology and Obstetrics, University Hospital, LMU Munich, Munich, Germany; 2 Department of Gynecology and Obstetrics, Erlangen University Hospital, Erlangen, Germany; 3 Department of Gynecology and Obstetrics, Medical Faculty and University Hospital, Heinrich-Heine University, Düsseldorf, Germany; 4 Department of Gynecology and Obstetrics, Heidelberg University Hospital, Heidelberg, Germany; 5 RoMed Klinikum Rosenheim, Rosenheim, Germany; 6 Gemeinschaftspraxis Lorenz / Hecker / Wesche, Braunschweig, Germany; 7 Department of Gynecology and Obstetrics, Ulm University Hospital, Ulm, Germany; 8 Laboratory for Experimental Radiology, Institute for Clinical Radiology, Ludwig-Maximilians-University Hospital, Marchioninistr. 15, 81377 Munich, Germany; 9 Department of Gynecology and Obstetrics, Medical University of Graz, Graz, Austria

**Keywords:** Early breast cancer, microRNA, Tumor marker, Circulating tumor cell, Immune system

## Abstract

**Background:**

microRNAs (miRNAs) are considered promising cancer biomarkers, showing high reliability, sensitivity and stability. Our study aimed to identify associations between whole blood miRNA profiles, presence of circulating tumor cells (CTCs) and clinical outcome in post-operative early breast cancer patients (EBC) to assess the utility of miRNAs as prognostic markers in this setting.

**Method:**

A total of 48 post-operative patients, recruited in frame of the SUCCESS A trial, were included in this retrospective study and tested with a panel of 8 miRNAs (miR-10b, −19a, − 21, − 22, −20a, − 127, − 155, −200b). Additional 17 female healthy donors with no previous history of cancer were included in the study as negative controls. Blood samples were collected at different time points (pre-adjuvant therapy, post-adjuvant therapy, 2 years follow up), total RNA was extracted and the relative concentration of each miRNA was measured by quantitative PCR and compared in patients stratified on blood collection time or CTC detection. Furthermore, we compared miRNA profiles of patients, for each time point separately, and healthy donors. CTCs were visualized and quantified with immunocytochemistry analysis. Data were analyzed using non-parametric statistical tests.

**Results:**

In our experimental system, miR-19a, miR-22 and miR-127 showed the most promising results, differentiating patients at different time points and from healthy controls, while miR-20a, miR-21 and miR-200b did not show any difference among the different groups. miR-10b and miR-155 were never detectable in our experimental system. With respect to patients’ clinical characteristics, we found a significant correlation between miR-200b and lymph node status and between miR-20a and tumor type. Furthermore, miR-127 correlated with the presence of CTCs. Finally, we found a borderline significance between Progression Free Survival and miR-19a levels.

**Conclusions:**

This pilot study suggests that profiling whole blood miRNAs could help to better stratify post-operative EBC patients without any sign of metastasis to prevent later relapse or metastatic events.

**Electronic supplementary material:**

The online version of this article (10.1186/s12885-018-4020-7) contains supplementary material, which is available to authorized users.

## Background

The impressive progress and success rate of treatment protocols and the enormous efforts made to improve prognosis of breast cancer (BC) have increased the 5-year-survival rate in the USA to over 90% [1]. However a number of patients finally succumb to the disease, due to the strong tendency of primary BC to spread with induction of incurable metastasis [2–6]. Detection of BC is mostly based on imaging [7, 8], but whilst this method can help to distinguish between benign and malignant lesions, it cannot provide any information about the presence of simultaneous hidden metastasis or the risk of relapse. In recent years, liquid biopsy has raised a lot of interest as a powerful method for cancer screening and monitoring. In particular, circulating tumor cells (CTCs) and cell-free nucleic acids such as microRNA (miRNA) have demonstrated independent prognostic and predictive relevance [9–13].

miRNAs are short non-coding single strand RNA sequences, ~ 21-25 nucleotides long, detectable in body fluids, cells and tissues [14]. By binding to the 3′ untranslated region of messenger RNAs, miRNAs can direct post-transcriptional repression, thereby fulfilling an important regulatory role in gene expression [14]. In recent years, miRNAs have been proposed as potential biomarkers for diagnosis, classification and treatment of different types of cancer, including BC [12, 15, 16]. Until now, most studies have focused on detection of miRNAs in body fluids such as serum and plasma, but recently whole blood miRNAs have also become attractive biomarkers in cancer pathogenesis, mainly in view of their possible role as immune system regulators [17–19]. miRNA are actively released in blood by cell secretion or passively as a consequence of cell lysis or apoptosis [20, 21] Several miRNAs are known to be involved in the development, maturation, differentiation and function of peripheral blood mononucleated cells (PBMC) including T and B lymphocytes and natural killer cells, as well as in antibody production and in inflammatory mediator release [22–24]. It has been shown that PBMCs go through several molecular changes already at the very early phases of neoplastic lesions [19] and a role of miRNAs in their differential expression has already been established [25]. Notably, immune system activation is not dependent on cancer burden. All of this suggests that the characterisation of whole blood miRNAs may be useful in the detection of primary malignancy or metastasis even in the early stages of their development [26, 27]. Therefore, the use of miRNAs as diagnostic tools for early detection of primary tumors or metastasis could be relevant in post-operative breast cancer patients to ensure timely treatment.

The aim of our study was to investigate alterations in whole blood miRNA levels in post-operative early BC (EBC) patients before and after therapy and at 2 years follow up, to evaluate their possible role as a novel class of biomarkers to better monitor patients with no sign of relapse or metastasis after surgery. We screened peripheral blood samples obtained from patients with no sign of metastasis at time of collection to measure the levels of 5 oncogenic (miR-10b, −19a, − 21, − 22, − 155) and 3 tumor suppressor (miR-20a, − 127 and -200b) miRNAs. These miRNAs have already been linked to carcinogenesis displaying multifunctional roles such as strong activators of proliferation, growth and invasion (miR-19a and miR-21) [20, 28], being involved in the induction of the epithelial-mesenchymal transition (EMT) and metastasis (miR-10b and miR-22) [29, 30], or on the contrary as inhibitors of cellular proliferation (miR-20a, miR-127 and miR-200b) [31–34]. Moreover, some of the miRNAs included in the panel (miR-19a, − 21, − 127 and 155) have been shown to be regulators of both innate and adaptive immune response [20, 31, 35, 36]. In this respect, the analysis of these miRNAs could offer the possibility to indirectly predict the development of metastasis as a consequence of a failure in the immune system reaction. The plausibility of using miRNAs as early surrogate markers for CTC detection was also evaluated as well as their possible role as predictors for clinical outcome.

## Methods

### Ethic statement

The patients’ cohort represented a subsample of the German multicenter open label phase III SUCCESS-A trial (NCT02181101) [9]. The study was approved by all the involved ethical boards and conducted in accordance with the Declaration of Helsinki [37]. All patients and healthy donors (HDs) provided written informed consent.

### Patients’ characteristics

A total of 48 EBC patients were included in this retrospective analysis [9]. All patients (mean age, years ± SD: 58.5 ± 11.0, range: 36-75) had histologically confirmed high risk BC (stages pT1-T4, pN0-N3, M0) according to standard clinical guidelines and underwent primary breast surgery. Blood samples were collected post-operative from EBC patients at three different time points: T0 (before chemotherapy, median number of days after surgery: 23); T1 (after chemotherapy, median numbers of days after surgery: 173); T2 (at 2 years follow up, median number of days after surgery: not available). Tumor classification was done according to the TNM guidelines [38]. Luminal cancer type A was defined as estrogen and/or progesterone receptor positive (ER^+^/PR^+^), human epidermal growth factor receptor-2 negative (HER2^−^) and grading (G) 1-2; luminal cancer type B was defined as ER^+^/PR^+^, HER2 positive or negative and G3; basal-like tumor was defined as ER^−^/PR^−^ and HER2^−^ (triple negative, TN); HER2-like tumor was defined as HER2 positive (HER2^+^). Patients’ clinical and histo-pathological details are summarized in Table 1. Additionally, 17 female HDs (mean age, years ± SD: 51 ± 9.7, range: 34-63) with no previous history of cancer were included in the study as negative controls.

**Table 1:** Patients’ and primary tumor’s characteristics

Total	48^a^
Mean age, years (SD)	58.5 (±11.0)
Range	36-75
Tumor size	
pT1a-c	14 (29.2%)
pT2-4	34 (70.8%)
Lymph node status	
Node negative	12 (25.0%)
Node positive (pN1-3)	35 (72.9%)
pNx	1 (2.1%)
Grading	
G1-2	27 (56.2%)
G3	21 (43.8%)
Estrogen receptor status ^b^	
ER positive	30 (62.5%)
ER negative	18 (37.5%)
Progesterone receptor status^c^	
PR positive	25 (52.1%)
PR negative	23 (47.9%)
HER2 status	
negative	36 (75.0%)
positive	11 (22.9%)
unknown	1 (2.1%)
Menopausal status	
Premenopausal	15 (31.3%)
Postmenopausal	33 (68.7%)
Primary operation	
Breast conservative	30 (62.5%)
Mastectomy	18 (37.5%)
Systemic therapy	
Chemotherapy-FEC^d^-DOC^e^	21 (43.8%)
Chemotherapy-FEC-DOC Gem^f^	27 (56.2%)

^a^Number of patients (percentage); ^b^*ER* estrogen receptor; ^c^*PR* progesterone receptor; ^d^*FEC* fluorouracil-epirubicin-cyclophosphamide; ^e^*DOC* docetaxel; ^f^*Gem* gemcitabine

### MicroRNA panel

In this exploratory study, we analyzed 8 miRNAs with oncogenic or tumor suppressive characteristics. The main properties (effects, targets and associated biological events) are summarized in Table 2. miRNAs were included in the panel on the basis of their relevance to breast cancer, induction of metastasis and association to immune system as reported in literature (Table 2).

**Table 2:** Oncogenic or tumor suppressor miRNAs analysed in the study: effect, targets and associated events

MicroRNA (family)	Effect	Identified target	Associated event	Reference
miR-10b	Oncogenic	HOXD10	Metastasis induction	[58]
miR-19a	Oncogenic	PTEN	Cell proliferation, Th1 immune response (innate immunity)	[20, 22, 59–61]
miR-20a	Tumor suppressor	E2F	Proliferation repression	[33]
miR-21	Oncogenic	TPM1, PDCD4, TIMP3, PTEN	Cell proliferation, migration, EMT, apoptosis inhibition, Treg cell activation	[20, 62–64]
miR-22	Oncogenic	miR-200, ERa, TET	Cell proliferation, EMT	[30, 65]
miR-127	Tumor suppressor	BCL-6	Proliferation, senescence, chemo- and radio-resistance, B cell activation	[31, 32]
miR-155	Oncogenic	STAT-3	Inflammation, B cell activation (innate/adaptive immunity)	[24, 36]
miR-200b	Tumor suppressor	E-cadherin, ZEB1, ZEB2	EMT, tumor growth, metastasis	[66, 67]

### Isolation of total RNA

Peripheral blood (3 mL) from patients and HDs was drawn directly in Tempus Blood RNA Tubes (ThermoFischer Scientific, Germany) to stabilize and isolate total RNA. After overnight shipment at room temperature, samples were frozen and stored at − 80 °C. Total RNA was isolated using the MagMAX™ for Stabilized Blood Tubes RNA Isolation Kit (ThermoFischer Scientific) according to the manufacturer’s instructions. In brief, frozen samples were thaw on ice for 30 min, centrifuged at 4500 g for 10 min at 4 °C, pellets were then treated with Tempus Proteinase and TURBO DNase and finally RNA purification was performed using RNA binding beads and a magnet stand. After removing the supernatant and washing twice with the provided washing buffer, beads were left drying at room temperature and total RNA was finally eluted in 40 μL elution buffer. The protocol allowed the recovery of approximately 3-25 μg total RNA. Quality of RNA was checked by 2% agarose gel electrophoresis (SYBR Safe E-Gel 2%, ThermoFischer Scientific) and RNA yield was determined spectrophotometrically (NanoDrop, Implen, Germany).

### miRNA analysis

Starting from total RNA, miRNAs were reverse-transcribed using the TaqMan MicroRNA Reverse Transcription Kit (ThermoFischer Scientific) and quantified using the TaqMan MicroRNA assay. Hydrolysis probes used in the study were purchased from ThermoFischer Scientific (hsa-miR-10b-3p 002315; hsa-miR-19a-3p 000395; hsa-miR-20a-3p 002437; hsa-miR-21-3p 002438; hsa-miR-22-3p 00398; hsa-miR-127-3p 000452; hsa-miR-155-3p 002287; hsa-miR-192-3p 002272; hsa-miR-200b-3p 002251). For each microRNA, 5 μL of total RNA (2 ng/μL) were mixed with 7 μL of RT reaction mix consisting of 0.15 μL 100 mM dNTPs (with dTTP), 1.00 μL MultiScribe Reverse Transcriptase (50 U/μL), 1.50 μL 10X Reverse Transcription Buffer, 0.19 μL RNase Inhibitor (20 U/μL) and 4.16 μL nuclease-free water. Specific microRNA RT primers (3 μL) were added to each reaction to a final volume of 15 μL. After incubation on ice for 5 min, reverse transcription (RT) was performed at 16 °C for 30 min, 42 °C for 30 min, 85 °C for 5 min (Mastercycler, Eppendorf, Germany). Quantitative reverse transcription-polymerase chain reaction (RT-qPCR) was performed immediately after RT; alternatively, cDNAs were stored at − 20 °C. In the negative controls, all specific RT primers were substituted with RNase/DNase-free water. RT-qPCR was run in a final volume of 20 μL reaction mix containing 1 μL 20X TaqMan Small RNA Assay, 10 μL 2X TaqMan Universal PCR Master Mix II no UNG (ThermoFischer Scientific), 7.67 μL nuclease-free water and 1.33 μL cDNA. All samples were run in triplicates; for each assay, no template controls were included to each plate. The plate was loaded into the 7500 Fast Real-Time PCR system (ThermoFischer Scientific) using the amplification standard mode (50 °C for 2 min, 95 °C for 10 min and 40 cycles at 95 °C for 15 s and 60 °C for 60 s). Relative expression of miRNAs was obtained using the eq. 2^-ΔCq^, where ΔCq = (Cq targeted miRNA) - (Cq reference miRNA) (Cq: quantification cycle) [39]. Each primer was tested separately to define the PCR amplification efficiency by means of calibration curves. Correlation coefficient (R^2^) and PCR efficiency calculated from slope were all between 0.97-0.99 and 82%-114%, respectively (Additional file 1: Table S1). miR-192 was used as reference miRNA to normalize the relative levels of the other miRNAs, as previously described [20]. miR-192 Cq mean values did not show any significant difference (always *p* > 0.05) in paired or unpaired groups analyzed with the Wilcoxon or the Kruskal-Wallis test, respectively. Furthermore, intra- and inter- group variation measured with the statistical algorithm Normfinder confirmed that miR-192 was stably expressed [40]. Since samples were collected in Tempus tubes which allow only a minimal variation in RNA extraction efficiency, no spike-in exogenous control was included.

### CTC isolation

Whole blood (23 mL) was collected in BD Vacutainer EDTA tubes (Becton Dickinsons, Germany) or CellSave tubes (Janssen Diagnostic, Raritan NJ, USA) and peripheral blood mononuclear cells (PBMCs) were isolated by density gradient (OncoQuick, Greiner BioOne, Germany). All mononuclear cells were collected from the interphase layer, washed two times in phosphate buffer saline (PBS) and finally spun down at 150 g for 5 min at room temperature (RT) on a SuperFrost® Plus glass slide (ThermoFischer Scientific). Cytospins were dried for 12-24 h at RT and then stained or stored at − 80 °C.

### CTC immune-detection and quantification

To detect CTCs, 2 cytospins per patient were stained with the pan-anti-cytokeratin monoclonal mouse A45-B/B3 antibody (dilution 1:100) (Micromet AG, Germany), which is directed against cytokeratin (CK) heterodimers 8/18 and 8/19 detectable in epithelial cells but not in white blood cells [41, 42]. CK is generally considered a valid tumor marker as shown by single cell genomic analysis of CK positive cells isolated by bone marrow of BC patients [29]. Antibody’s quality and specificity were controlled using the cytokeratin positive human breast adenocarcinoma cell line MCF-7 (ATCC® HTB-22™). The primary antibody was labelled using the DAKO alkaline phosphatase-anti-alkaline phosphatase (APAAP) detection system, with the Z0259 antibody combined with new fuchsin staining as secondary antibody (DakoCytomation, Denmark). The human breast cancer cell line BT-20A (ATCC® HTB-19™) was used as positive control (data not shown). The murine antibody clone MOPC21 (Sigma-Aldrich Chemie GmbH, Germany) was used as IgG1 kappa isotype negative control to test the antibody reaction specificity (data not shown). After staining, slides were screened by two independent investigators under a standard bright field Axiophot microscope (Carl Zeiss, Germany) equipped with a 40 fold magnification objective. Few samples were not analyzable (n.a.) due to technical failures. Patients were classified as CTC positive when at least one CTC was detected. Only immunocytochemically positive cells with a moderate to strong signal intensity and no hematopoietic characteristics were defined as CTCs.

### Statistical analysis

GraphPad Prism version 6.00 for Windows (GraphPad Software, La Jolla CA, USA) was used for running the statistical analysis. The non-parametric Mann-Whitney U test was used to compare miRNA levels between different patient groups (the Kruskal-Wallis test was used in case of more than two groups) and the Wilcoxon matched-pairs signed rank test was used to compare miRNA levels obtained from the same patients at different time points. Receiving Operator Characteristics (ROC) curves gave the diagnostic power of whole blood miRNA levels; areas under the curves (AUC) were calculated for each case and were considered excellent between 0.9 and 1.0, good between 0.8 and 0.9, fair between 0.7 and 0.8, poor between 0.6 and 0.7 and failed between 0.5 and 0.6. Overall survival (OS) and progression free survival (PFS) were analyzed using the Kaplan-Meier method and survival estimates in different groups were compared using the log-rank test. For survival analysis, high and low miRNA levels were defined as being above or below the mean values of each miRNA in HD plus 1 standard deviation (SD). Two-sided *p*-values below 0.05 were considered statistically significant and no adjustment of the significance level for multiple testing was performed.

## Results

### Comparison of miRNA levels between EBC patients and healthy donors

The relative amounts of the 5 oncogenic (miR-10a, −19a, − 21, − 22, − 155) and the 3 tumor suppressor (miR-20a, − 127 and -200b) miRNAs were measured in patients’ whole blood drawn before adjuvant therapy (T0, *n* = 47), after adjuvant therapy (T1, *n* = 14) and at 2 years follow up (T2, *n* = 17), and compared, for each time point separately, to those found in healthy donors (HDs) (*n* = 17). miR-10b and miR-155 were never detectable in any of the samples analyzed, including those withdrawn from healthy donors, although preliminary tests run in cell lines indicated an adequate amplification efficiency. The level of these miRNAs was most probably below the detection limit; further work will be necessary in order to confirm this hypothesis. Results are therefore reported for the six remaining miRNAs only.

**Fig. 1:**
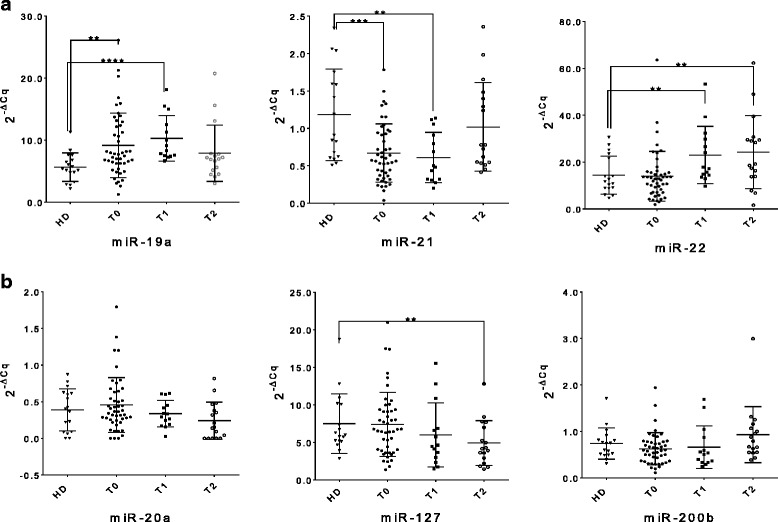
Whole blood miRNA levels in patients at T0, T1 and T2. The dot plots show the relative levels of oncogenic miR-19a, − 21 and − 22 (**a**), and tumor suppressor miR-20a, − 127 and -200b (**b**). Comparisons were performed (for each time point separately) using the Mann-Whitney U test and the corresponding *p*-values of significant differences are indicated in the graphs (^*^p ≤ 0.05; ^**^p ≤ 0.01; ^***^p ≤ 0.001; ^****^p ≤ 0.0001). (HD: healthy donor; T0: before adjuvant therapy; T1: after adjuvant therapy; T2: at 2 years follow up)

Compared to HDs, patients showed higher levels of miR-19a at T0 (median 7.70 vs. 5.36, *p* = 0.004) and T1 (median 8.74 vs. 5.36, *p* < 0.0001), lower levels of miR-21 at T0 (median 0.57 vs. 0.91, *p* = 0.001) and T1 (median 0.49 vs. 0.91, *p* = 0.004), and higher levels of miR-22 at T1 (median 17.54 vs. 11.72, *p* = 0.012) and at T2 (median 19.20 vs 11.72, *p* = 0.034) (Fig. 1a). Among the tumor suppressors, only miR-127 was significantly down-regulated at T2 (median 5.61 vs 3.78, *p* = 0,028; Fig. 1b). No other significant difference between patients and HDs was observed. ROC curve analysis confirmed that miR-19a (T0, AUC = 0.732, *p* = 0.004; T1, AUC = 0.8908, *p* = 0.0002), miR-21 (T0, AUC = 0.7572, *p* = 0.001; T1, AUC = 0.7983, *p* = 0.004), miR-22 (T1, AUC = 0.7647, *p* = 0.012; T2, AUC = 0.7128, *p* = 0.034) and miR-127 (T2, AUC = 0.7197, *p* = 0.028) could differentiate patients from HDs at the different time points.

### Comparison of miRNA levels in the same EBC patients at different time points

**Fig. 2:**
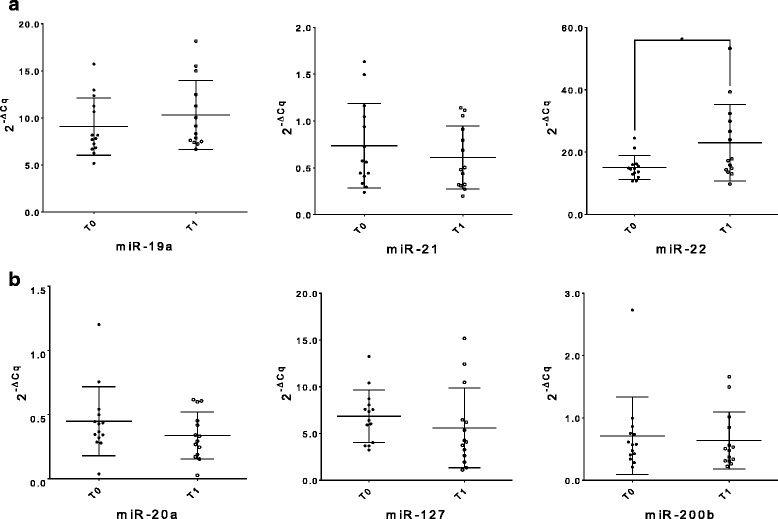
Whole blood miRNA levels inter-differentiating the same patient group at T0 and T1. The dot plots show relative levels of the different oncogenic miR-19a, − 21 and − 22 (**a**) and tumor suppressor miR-20a, − 127 and -200b (**b**). The differences were calculated using the Wilcoxon test; only the corresponding significant *p*-values are indicated in the graphs (^*^p ≤ 0.05). (T0: before adjuvant therapy; T1: after adjuvant therapy)

**Fig. 3:**
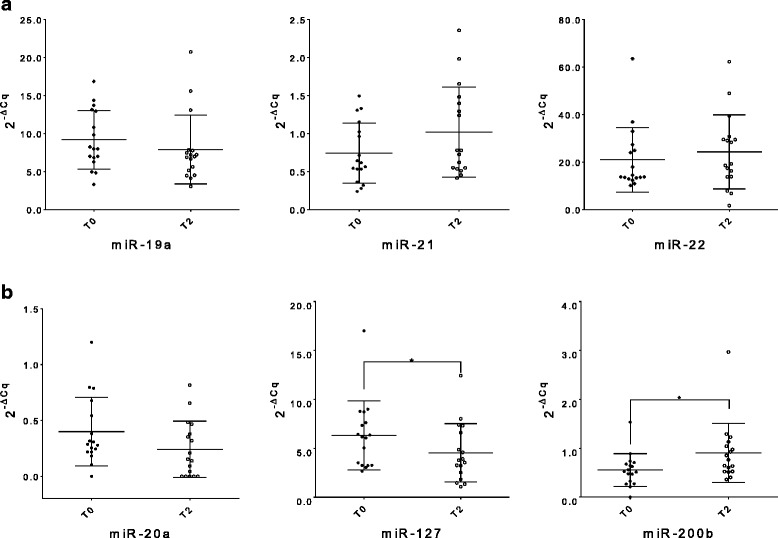
Whole blood miRNA levels inter-differentiating the same patient group between T0 and T2. The dot plots show relative levels of the different oncogenic miR-19a, − 21 and − 22 (**a**) and tumor suppressors miR-20a, − 127 and -200b (**b**). The differences were calculated using the Wilcoxon test; only the corresponding significant *p*-values are indicated in the graphs (^*^p ≤ 0.05). (T0: before adjuvant therapy; T1: after adjuvant therapy)

miRNA levels were measured and compared within patients according to the time of the blood collection. We found no significant difference in the miRNA levels measured in each patient before (T0) and after (T1) chemotherapy. The only exception was given by the oncogenic miR-22, which showed a significant upregulation at T1 (*n* = 14; *p* = 0.028) (Fig. 2). A pairwise miRNA comparison in the same patients at T0 and T2 (2 years follow up) revealed a significant downregulation of the tumor suppressor miR-127 (n = 14; *p* = 0.041), while the tumor suppressor miR-200b showed a minimal however significant increase (*n* = 14; *p* = 0.049) (Fig. 3). Pairwise comparisons between miRNA levels obtained at T1 and T2 did not show any significant difference among miRNA levels (data not shown). ROC curve analysis confirmed that miR-22 (AUC = 0.79, *p* = 0.002), miR-127 (AUC = 0.98, *p* < 0.0001) and miR-200b (AUC = 0.69, *p* = 0.01) could differentiate the same patients at the different time points.

### Association of miRNA levels with tumor characteristics and tumor subtypes at baseline

**Fig. 4:**
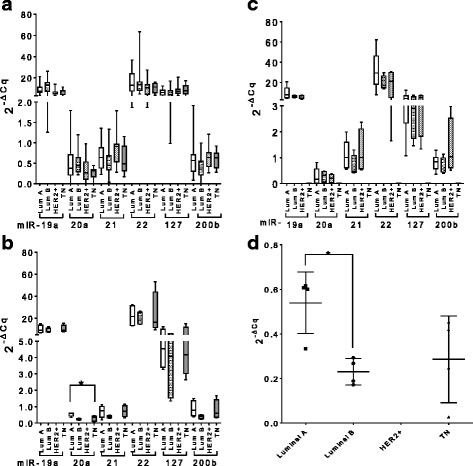
Whole blood miRNA levels in patients with different breast cancer subtypes at different time points. The box plots show relative levels of the different oncogene miRNAs and tumor suppressors miRNAs in the different patients subgroups analyzed at T0 (**a**), T1 (**b**) and T2 (**c**). The differences were calculated using the Kruskal-Wallis test. Only miR-20a showed a significant difference between the four cancer types after therapy. miR-20a was further analyzed using the Mann-Whitney U test to compare the four different cancer subtypes. A significant difference between luminal cancer type A and B is indicated in the dot plot (**d**) (^*^p ≤ 0.05). (T0: before adjuvant therapy; T1: after adjuvant therapy; T2: at 2 years follow up; Lum A: luminal breast cancer type A; Lum B: luminal breast cancer type B; HER2: HER2-like tumor; TN: triple negative breast cancer)

The median level of each miRNA assessed at T0 was also compared between patients’ sub-groups with different tumor sizes (pT1 vs. pT2-3), nodal involvement (pN0 vs pN1-3) and grading (G1-2 vs G3) (Table 3). The tumor suppressor miR-200b showed a significant downregulation (median 0.73 vs 0.47, *p* = 0.003) in pN1-3 patients (positive for lymph node metastasis) (*n* = 35) as compared to pN0 patients (negative for lymph node metastasis) (*n* = 12), while all the other miRNAs could not differentiate the patients according to the histopathological characteristics. A comparison between cancer subtypes (luminal cancer type A, luminal cancer type B, basal like and HER2-like) and the corresponding hormonal status with the relative levels of each miRNA at the three time points T0, T1 and T2 was also performed (Fig. 4). No significant differences were found with the only exception of miR-20a at T1 (panel B): higher levels were measured in patients with luminal cancer type A (*n* = 4) with respect to patients with luminal cancer type B (*n* = 4) (median 0.60 vs 0.22, *p* = 0.028, AUC = 1, *p* = 0.020) (panel D). The results were nevertheless obtained with an extreme small patient number therefore they should be considered with caution; further experiments with a larger cohort of patients will be performed to validate these preliminary data.

**Table 3:** Comparisons of baseline miRNA levels in early breast cancer patients according to tumor characteristics. The differences were calculated using the Mann-Whitney U test and the corresponding *p*-values are indicated in the table (significant *p*-values are indicated in bold). (pT: tumor grade; pN: lymph node status; G: tumor grade)

Tumor characteristics	miR-19a	miR-20a	miR-21	miR-22	miR-127	miR-200b
Tumor stage						
pT1 vs. pT2-4	0.9345	0.8602	0.3771	0.9719	0.7549	0.1049
Lymph node status						
pN0 vs. pN1-3	0.1367	0.8757	0.2502	0.4050	0.5517	**0.0032**
Grading						
G1-2 vs. G3	0.1950	0.7736	0.8899	0.6164	0.8438	0.2286

### Comparison of miRNA levels at baseline in CTC-positive and CTC-negative EBC patients

**Fig. 5:**
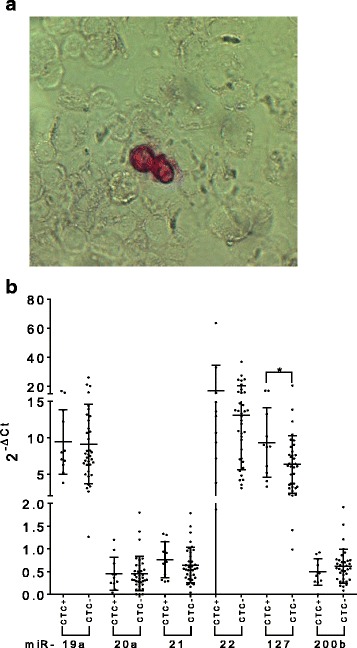
Whole blood miRNA levels comparison between CTC^+^ and CTC^−^ patients. Cytokeratin positive circulating tumor cells (CTCs) (in red) stained with the APAAP immunodetection system (**a**). Dot plots show relative levels of oncogenic miR-19a, − 21 and − 22 and of tumor suppressors miR-20a, − 127 and -200b in the different patients’ subgroups analyzed at T0 (before adjuvant therapy). Only miR-127 showed a significant difference between CTC^+^ and CTC^−^ patients. The differences were calculated using the Mann-Whitney U test (AUC = 0.70; *p* = 0.043) (^*^p ≤ 0.05) (**b**)

Pre-chemotherapy miRNA levels were compared between patients found CTC positive (*n* = 11) or CTC negative (*n* = 36) according to the immune-cytochemical analysis. Patients were classified as CTC positive when at least one cytokeratin positive cell was detected (Fig. 5a). CTC positive patients could be differentiated from CTC negative patients by the tumor suppressor miR-127, which showed a slight but nevertheless significant higher level in CTC positive patients (median 8.77 vs 6.10, *p* = 0.0424) (Fig. 5b). ROC curve analysis confirmed these findings (AUC = 0.70, *p* = 0.043). None of the other miRNAs could differentiate CTC positive from CTC negative patients.

#### Association between miRNA levels at baseline and clinical outcome

**Fig. 6:**
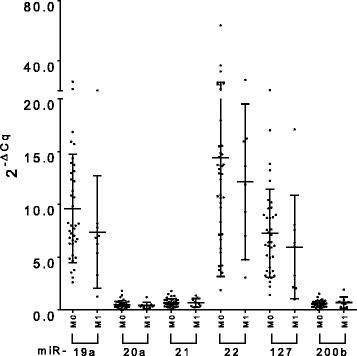
Whole blood miRNA levels in patients at T0. The dot plots show the relative levels of the different miRNAs for patients that developed metastasis and those that did not. The miRNA levels were compared between M0 and M1 patients using the Mann-Whitney U test; no significant differences were found. (M0: metastasis negative; M1: metastasis positive; T0: before adjuvant therapy)

miRNAs released in circulation in the early phases of tumor development originate from the primary tumor as well as from the cellular component of the immune system [43]. We hypothesized that miRNAs could predict the possible development of metastasis at later time points, possibly mirroring the immunological reaction to the primary tumor. Therefore, in order to unravel a miRNA prognostic relevance in metastasis development already in EBC patients with no sign of metastasis, we compared the miRNA levels at T0 of those patients who did not develop metastasis (M0, *n* = 38) with those who did develop metastasis during the follow up period (M1, *n* = 9). We could detect a higher median level of miR-19a in M0 compared to M1 patients, however the difference was not significant (median 7.981 vs. 6.816, *p* = 0.175). In the same way, no other miRNA showed any significant difference between the two groups (Fig. 6).

**Fig. 7:**
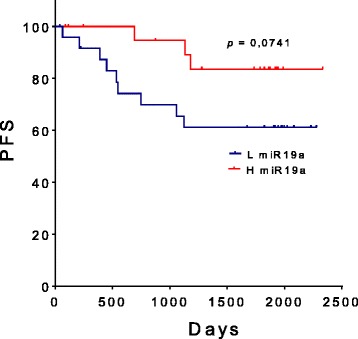
Progression-free survival (PFS) according to whole blood miR19a levels. High (H) and low (L) miR19a levels were defined as being above or below the mean values of miR-19a in HDs plus 1standard deviation (SD). PFS was analyzed using the Kaplan-Meier method and survival estimates in different groups were compared using the log-rank test

The relative levels of miRNAs at T0 were also evaluated with respect to their predictive role in patients’ clinical outcome in terms of PFS and OS. Patients were grouped in low and high expressors according to a cut off value corresponding to the mean values of each miRNA plus 1SD in HDs. Only the levels of miR-19a reached borderline significance for PFS (HR = 3.091, 95% confidence interval [CI] =0.904-8.709, *p* = 0.074) (Fig. 7). No other miRNA did show any significant (or close to significant) variation with respect to PFS or OS.

## Discussion

The role of miRNA as non-invasive biomarkers has been proposed in different type of diseases including cancer. miRNA are considered ideal markers because of their high stability in extreme conditions such as low pH or high temperatures [44]. Their presence in body fluids other than plasma and serum, such as urine [45], saliva [46] and pancreatic juice [47] has been shown to have a solid diagnostic value [48]. Independent studies have also established the value of whole blood miRNA profiling in early phases of cancer development [26, 27, 49, 50]. In this study the relative level of a panel of miRNAs with oncogenic or tumor suppressor properties, in part also acting as immune system modulators, was measured in post-operative EBC patients and compared to healthy donors or within patient sub-groups. We showed that the levels of miR-19a, miR-21, miR-22 and miR-127 could significantly discriminate post-operative non-metastatic EBC patients from healthy donors before and/or after adjuvant chemotherapy and at 2 years follow up, indicating their potential diagnostic value. For both miR-19a and miR-21, we did not detect any difference between their relative levels measured before and after therapy, suggesting that changes in the expression profile were independent of treatment. On one hand, miR-21 was significantly downregulated at T0. This result was somehow surprising since miR-21 is usually showing higher levels in serum or plasma of BC patients. However, since we monitored the miRNA level in whole blood, a drastic post-operative decrease in miR-21 levels could indicate a deregulation of the immune and/or inflammatory process, possibly enhancing the neoplastic disease and promoting proliferation and migration [35, 51]. In other words the relative upregulation of miR-21 in cancer cells could have been “diluted” by the higher number of PBMC characterized by an evident miR-21 downregulation. Further studies will be nevertheless necessary to confirm this hypothesis. miR-19a, on the other hand, showed significantly higher mean values before and after therapy. Since miR-19a expression is higher in activated lymphocytes [17, 20, 52], we reasoned that a better prognosis as suggested by survival curve analysis in post-operative patients could have been influenced by a stronger early immune response. With regard to the tumor suppressor miR-127, we found no significant difference between EBC patients and healthy donors at the earlier time points, while a significant lower level was detected at 2-year follow-up. In addition, miR-127 levels measured repeatedly for the same patients decreased significantly from T0 to T2. miR-127 has been shown to be downregulated in BC tissue compared with corresponding healthy tissue and to correlate with an advanced clinical stage and metastasis development [32]. The principal target of miR-127 is the proto-oncogene BCL6 [31], which plays a direct role in survival, proliferation and differentiation of B lymphocytes [53]. Consequently, lower levels of miR-127 in whole blood might indicate a parallel activation of B-cells at a later time point. Surprisingly, miR-127 gave a different result when the patients were stratified according to the presence or absence of CTCs at T0. In this case, miR-127 displayed to be upregulated in CTC positive patients compared to CTC negative patients. Other studies have already shown a correlation between CTCs and upregulation of miRNAs with tumor suppressor activity as is the case with the miR-200 family [54]. Over-expression of miR-200 s supports the metastatic potential of CTCs inducing the mesenchymal-epithelial-transition (MET), an essential step for starting and developing new metastases. The fourth miRNA which showed significant differences between EBC patients and healthy controls was miR-22, a potent activator of EMT and cell proliferation. miR-22 was upregulated in patients after chemotherapy and at 2-year follow up, a finding which might suggest a selective therapeutic pressure favoring the development of aggressive chemotherapy-resistant tumor cells and possibly micrometastasis with mesenchymal characteristics. Finally, miR-20a and miR-200b, although always detectable, failed to significantly differentiate EBC patients from healthy controls or between patients at different time points, and therefore showed no prognostic value. However, both miRNAs showed some grade of correlation with the primary tumor’s characteristics. miR-20a could differentiate post-operative patients with less aggressive luminal A from those with more aggressive luminal B primary cancer, while miR-200b showed lower mean values in pN1-3 patients compared to pN0 patients. Further studies will be necessary to confirm these preliminary findings.

Although our results are promising, some limitations in this work must be mentioned and addressed in future experimental work. Sampling has been performed retrospectively and the size of the patient’s cohort should be expanded to unravel patients’ subgroups or treatment regimens for which miRNAs could prove to be potential predictive markers. In addition, SUCCESS A clinical trial protocol missed an early pre-operative time point for blood collection; therefore we cannot completely exclude that variations in miRNAs levels are a consequence of surgery after systemic immune response. Most of the patients (62.5%) received nevertheless breast conserving surgery, a less invasive and less stressing procedure with respect to mastectomy and blood samples were collected several days (between 23 and 173) post-surgery, a time frame long enough to assume that post-operative immune functions had reverted to physiological conditions [27, 43] and that inflammation associated miRNAs were disappeared [27, 55]. Furthermore, miRNAs have been isolated not only from PBMCs but from whole cellular blood fractions, including platelets, granulocytes and red blood cells. Further studies will be necessary to establish if contamination from cells other than PBMCs can negatively affect the analysis and should be eliminated in some way [56]. In addition, due to the relative small cohort size, the data must be further validated with a larger number of cases allowing a more robust statistical testing. Finally the detection of CTCs was based on an immunostaining method and not on the FDA-cleared CellSearch® system, till now considered the gold standard for CTC isolation and enumeration [57].

## Conclusion

In conclusion, although this work is exploratory and a first hint pointing the way to future studies based on larger cohorts, nevertheless it suggests that analysis of whole blood miRNAs, linked to different physiological, immunological and pathological conditions, could help to better stratify post-operative BC patients, thereby supporting tailored therapies with a clear benefit to patient’s management.

## Additional file

Additional file 1: Table S1. Primer PCR efficiency calculate from slope and correlation coefficients (r^2^ values). (DOCX 11 kb)

## Abbreviations

BC: Breast cancer
CTC: Circulating tumor cell
DTC: Disseminated tumor cell
EBC: Early breast cancer
EMT: Epithelial-mesenchymal-transition
ER: Estrogen receptor
G: Tumor grade
HD: Healthy donor
miRNAs: microRNAs
OS: Overall survival
PBMC: Peripheral blood mononucleated cell
PFS: Progression free survival
pN: Lymph node status
PR: Progesterone receptor
pT: Tumor grade
