# Supplementary material for: Whole blood microRNAs as potential biomarkers in post-operative early breast cancer patients
Source: BMC Cancer. 2018 Feb 6;18:141. doi: 10.1186/s12885-018-4020-7 (PMC5802058; doi:10.1186/s12885-018-4020-7)
Supplement: Additional file 1: Table S1. — Primer PCR efficiency calculate from slope and correlation coefficients (r2 values). (DOCX 11 kb) [file 12885_2018_4020_MOESM1_ESM.docx]

Table S1. Primer PCR efficiency calculate from slope and correlation coefficients (r^2^ values).

| primer | Efficiency calculated from slope (%) | r^2^ values |
| --- | --- | --- |
| miR-192-3p | 114 | 0.97 |
| hsa-miR-10b-3p | 82 | 0.99 |
| hsa-miR-19a-3p | 101 | 0.99 |
| hsa-miR-20a-3p | 98 | 0.99 |
| hsa-miR-21-3p | 92 | 0.99 |
| hsa-miR-22-3p | 91 | 0.99 |
| hsa-miR-127-3p | 90 | 0.98 |
| hsa-miR-155-3p | 96 | 0.98 |
| hsa-miR-200b-3p | 107 | 0.99 |
